# Clinical features and outcomes of abdominal tuberculosis in southeastern Korea: 12 years of experience

**DOI:** 10.1186/s12879-018-3635-2

**Published:** 2018-12-27

**Authors:** Jin-Kyu Cho, Young Min Choi, Sang Soo Lee, Hye Kyong Park, Ra Ri Cha, Wan Soo Kim, Jin Joo Kim, Jae Min Lee, Hong Jun Kim, Chang Yoon Ha, Hyun Jin Kim, Tae Hyo Kim, Woon Tae Jung, Ok Jae Lee

**Affiliations:** 1 Department of Surgery, Gyeongsang National University Hospital, 15, Jinju-daero 816 beon-gil, Jinju-si, 52727 Gyeongnam Republic of Korea; 2 Department of Internal Medicine, Gyeongsang National University School of Medicine and Gyeongsang National University Hospital, 15, Jinju-daero 816 beon-gil, Jinju-si, Gyeongnam 52727 Republic of Korea; 3 Institute of Health Sciences, Gyeongsang National University, Jinju, Republic of Korea; 4 Department of Internal Medicine, Gyeongsang National University Changwon Hospital, Changwon, Republic of Korea

**Keywords:** Tuberculosis, Abdomen, Extra-pulmonary, Luminal, Peritoneal

## Abstract

**Background:**

Abdominal tuberculosis (TB) is an uncommon form of infection with *Mycobacterium tuberculosis* in Korea. In this study, we aimed to highlight the clinical features, diagnostic methods, and outcomes of abdominal TB over 12 years in Southeastern Korea.

**Methods:**

A total of 139 patients diagnosed as having abdominal TB who received anti-TB medication from January 2005 to June 2016 were reviewed. Among them, 69 patients (49.6%) had luminal TB, 28 (20.1%) had peritoneal TB, 7 (5.0%) had nodal TB, 23 (16.5%) had visceral TB, and 12 (8.6%) had mixed TB.

**Results:**

The most frequent symptoms were abdominal pain (34.5%) and abdominal distension (21.0%). Diagnosis of abdominal TB was confirmed using microbiologic and/or histologic methods in 76 patients (confirmed diagnosis), while the remaining 63 patients were diagnosed based on clinical presentation and radiologic imaging (clinical diagnosis). According to diagnostic method, frequency of clinical diagnosis was highest in patients with luminal (50.7%) or peritoneal (64.3%) TB, while frequency of microscopic diagnosis was highest in patients with visceral TB (68.2%), and frequency of histologic diagnosis was highest in patients with nodal TB (85.2%). Interestingly, most patients, except those with nodal TB, showed a good response to anti-TB agents, with 84.2% showing a complete response. The mortality rate was only 1.4% in the present study.

**Conclusions:**

Most patients responded very well to anti-TB therapy, and surgery was required in only a minority of cases of suspected abdominal TB.

## Background

Abdominal tuberculosis (TB) is defined as infection of the gastrointestinal tract, peritoneum, abdominal solid organs, and/or abdominal lymphatics with *Mycobacterium tuberculosis* [1]. Abdominal TB constitutes approximately 12% of extrapulmonary TB cases and 1 to 3% of total TB cases [1, 2]. Abdominal TB is one of the most common forms of extrapulmonary TB [3]. Abdominal TB is relatively rare, but it is recognized that abdominal TB is increasing in both developing and developed countries [4–8]. Abdominal TB accounts for approximately 4% of all TB cases in Korea [9]. Diagnosis of abdominal TB is often overlooked and delayed due to lack of specific symptoms and no specific diagnostic test. A high index of suspicion is necessary for early diagnosis of abdominal TB; however, it remains a considerable diagnostic dilemma and can mimic many other diseases, such as Crohn’s disease, abdominal lymphoma, and malignancy of the abdominal organs.

Abdominal TB can usually be classified into 4 forms: luminal, peritoneal, nodal, and visceral involving the intra-abdominal solid organs [10]. The most common forms are luminal (ileocecal area) and peritoneal [11]. The modes of infection of abdominal TB include swallowing infected sputum, ingestion of bacilli from infected milk products or meat, hematogenous spread from a lung focus, spread via lymphatics from infected lymph nodes, and contiguous spread from adjacent organs [12]. The clinical presentation of abdominal TB depends on the site of infection. Abdominal pain, diarrhea, bleeding from the luminal tract, intestinal obstruction, fever, and weight loss are frequent features of intestinal TB; ascites and abdominal distension are common manifestations of peritoneal TB [8]. Diagnosis of abdominal TB may also vary depending on the site of infection [13]. Colonoscopy is useful in patients suspected of having intestinal TB, while laparoscopy and biopsy are more useful in peritoneal TB; although ascitic fluid analysis is more accessible, its acid-fast bacilli (AFB) culture has a low sensitivity.

The aim of the present study was to evaluate the clinical features, diagnostic methods, and outcomes of abdominal TB, including luminal, peritoneal, nodal, visceral, and mixed TB.

## Methods

### Study populations

Between January 2005 and June 2016, a total of 139 consecutive adult patients aged > 18 years diagnosed as having abdominal TB began treatment with anti-TB drugs at Gyeongsang National University Hospital, located on the southeast coast of Korea. Age, sex, body mass index, alcohol consumption, history of TB, history of malignancy, TB infection site, date of anti-TB drug prescription, regimen of anti-TB drugs, laboratory data, underlying disease, clinical features, diagnostic method, and clinical outcome were reviewed. The present study was approved by the Institutional Review Board of the Gyeongsang National University Hospital.

### Definitions and classification

Abdominal TB was defined as infection of the luminal tract, peritoneum, intra-abdominal lymph nodes, and/or intra-abdominal solid-organs with *M. tuberculosis*. Diagnosis of abdominal TB was based on: (1) positive AFB smear or culture from ascites, urine, or biopsy specimen (microbiologic diagnosis); (2) demonstration of caseating granulomas on biopsy specimen (histologic diagnosis); (3) typical presentation and good response to anti-TB agents (clinical diagnosis); or (4) high index of suspicion in susceptible patients and good response to anti-TB agents (clinical diagnosis). When peritoneal TB could not be diagnosed by biopsy, the high ascitic adenosine deaminase (> 33 IU/L) criteria was used for clinical diagnosis. Confirmed diagnosis was defined as microbiologic or histologic evidence of *M. tuberculosis* [14].

Patients were classified into 5 groups according to site of TB infection: (1) luminal, (2) peritoneal, (3) nodal, (4) visceral, and (5) mixed. Luminal TB was further divided into esophageal, gastric, duodenal, jejunal, ileocecal, and colorectal. Peritoneal TB was divided into 3 types [10, 15]: (1) the wet ascitic type was associated with large amounts of free or loculated ascites; (2) the fixed fibrotic type was associated with involvement of the omentum and mesentery and was characterized by presence of bowel loop entanglement; and (3) the dry plastic type was characterized by peritoneal and mesenteric thickening with caseous nodules and presence of adhesions. To avoid confusion, we classified peritonal TB into two types, the wet and dry type (fixed fibrotic and dry plastic type). Nodal TB was divided into mesenteric, porta hepatitis, celiac axis, peripancreatic, and combined. Visceral TB was divided into hepatic, splenic, genitourinary, adrenal, and combined.

### Clinical outcomes

Abdominal TB outcomes were classified as follows [16]: (1) complete response: resolution of symptoms, disappearance of AFB on smear or culture, disappearance of tuberculous granulomas, and disappearance or healing of active tuberculous lesions on relook colonoscopy; (2) partial response: resolution of symptoms and partial disappearance of tuberculous lesions at end of treatment; (3) no response: persistence of symptoms, persistence of AFB on smear or culture, persistence of tuberculous granulomas, and persistence of active tuberculous lesions on relook colonoscopy at end of treatment; (4) lost to follow up: treatment interrupted for ≥2 consecutive months; (5) death: death from any cause during treatment; (6) recurrence: colonoscopic or radiologic documentation of recurrent lesions after a complete response had been achieved; and (7) transfer out: transferred to another reporting and recording unit.

### Statistical analysis

All analyses were performed using PASW version 18 (SPSS Inc., Chicago, IL, USA). Continuous variables were expressed as median (interquartile range). Intergroup differences in quantitative data were measured using the Mann-Whitney *U* test, while the Fisher exact test was used for qualitative data. All analyses were 2-sided and a *P* value of < 0.05 was considered statistically significant.

## Results

### Patient characteristics

**Fig. 1:**
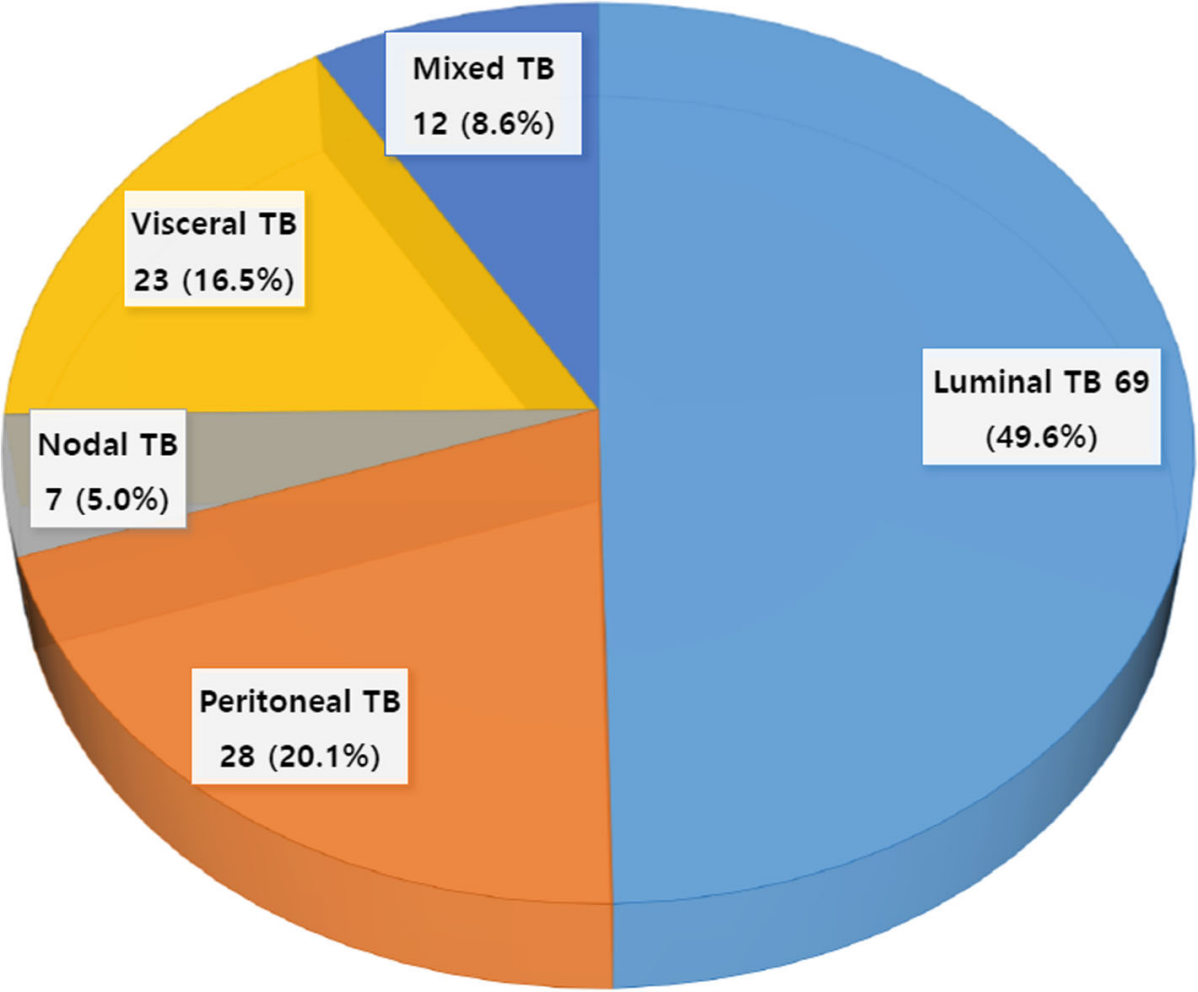
Sites of abdominal tuberculosis (TB)

A total of 139 patients (72 male, 51.8%) with a median age of 47.0 years were diagnosed has having abdominal TB. Among them, 69 patients (49.6%) were diagnosed with luminal TB, 28 (20.1%) with peritoneal TB, 7 (5.0%) with nodal TB, 23 (16.5%) with visceral TB, and 12 (8.6%) with mixed TB (Fig. 1). The ileocecum (67.1%) was the most frequently involved site in patients with luminal TB. Wet ascitic type (75.7%) was the most common type in patients with peritoneal TB. Genitourinary tract (69.0%) and porta hepatitis (35.7%) were the most frequently involved sites in patients with visceral and nodal TB, respectively (Table 1). The demographic characteristics of the patients are presented in Table 2. There were no significant differences in sex, body mass index, chronic hepatitis B, HIV infection, alcohol consumption, liver cirrhosis, end-stage renal disease, history of malignancy, history of TB, or anemia at diagnosis among the 5 study groups. The mixed TB group was significantly younger (median age, 32.0 years) than the other 4 groups. The rate of history of TB was significantly higher in the visceral TB group (21.7%) than in the peritoneal TB group (0%).

**Table 1:** Site of involvement in abdominal tuberculosis (*n* = 139)

Site	N (%)
Luminal TB	
Esophageal	3 (3.9%)
Stomach	0 (0%)
Duodenal	3 (3.9%)
Jejunal	7 (9.2%)
Ileocecal	51 (67.1%)
Colorectal	12 (15.8%)
Peritoneal TB	
Wet ascitic type	28 (75.7%)
Dry ascitic type	9 (24.3%)
Nodal TB	
Mesenteric	2 (14.3%)
Porta hepatis	5 (35.7%)
Along the celiac axis	1 (7.1%)
Peripancreatic	2 (14.3%)
Retroperitoneal	1 (7.1%)
Combined	3 (21.4%)
Visceral TB	
Hepatic	3 (10.3%)
Splenic	4 (13.8%)
Genitourinary	20 (69.0%)
Adrenal	1 (3.4%)
Combined	1 (3.4%)
Mixed TB	
Luminal and peritoneal	4 (33.3%)
Luminal and nodal	1 (8.3%)
Luminal and peritoneal, and nodal	2 (16.7%)
Peritoneal and visceral	2 (16.7%)
Nodal and visceral	2 (16.7%)
Peritoneal and visceral, and nodal	1 (8.3%)

TB: tuberculosis

Data are presented as the median (interquartile range) for continuous data and percentages for categorical data

**Table 2:** Demographic characteristics of 139 patients with abdominal tuberculosis

Characteristic	All patients (*n* = 139)	Luminal TB (*n* = 69)	Peritoneal TB (*n* = 28)	Nodal TB (*n* = 7)	Visceral TB (*n* = 23)	Mixed TB (*n* = 12)
Age	47.0 (32.0–58.0)	48.0 (38.5–58.5)	52.5 (27.3–63.0)	53.0 (27.0–57.0)	46.0 (33.0–56.0)	32.0 (25.5–45.8) ^d, g, j^
Male sex	72 (51.8%)	36 (52.2%)	18 (64.3%)	3 (42.9%)	9 (39.1%)	6 (50%)
BMI (m/kg2)	21.80 (19.56–24.53)	21.3 (19.6–24.1)	21.6 (19.3–24.5)	23.7 (20.1–27.8)	20.1 (22.8–25.3)	20.8 (18.0–22.8)
Chronic hepatitis B	10 (7.2%)	5 (7.2%)	2 (7.1%)	0 (0%)	2 (8.7%)	1 (8.3%)
Chronic hepatitis C	3 (2.2%)	1 (1.4%)	0 (0%)	2 (28.6%) ^b, e, h^	0 (0%)	0 (0%)
HIV-infection	2 (1.4%)	0 (0%)	0 (0%)	0 (0%)	1 (4.3%)	1 (8.3%)
Alcohol > 40 g/day	16 (11.5%)	6 (8.7%)	6 (21.4%)	0 (0%)	2 (8.7%)	2 (16.7%)
Cirrhosis	3 (2.2%)	2 (2.9%)	0 (0%)	0 (0%)	0 (0%)	1 (8.3%)
Diabetes	9 (6.5%)	3 (4.3%)	3 (10.7%)	1 (14.3%)	2 (8.7%)	0 (0%)
ESRD or CAPD	8 (5.8%)	3 (4.3%)	3 (10.7%)	1 (14.3%)	1 (4.3%)	0 (0%)
History of malignancy	7 (5.0%)	3 (4.3%)	2 (7.1%)	0 (0%)	2 (8.7%)	0 (0%)
History of TB	13 (9.4%)	7 (10.1%)	0 (0%)	1 (14.3%)	5 (21.7%) ^f^	0 (0%)
Anemia at diagnosis	46 (33.1%)	21 (30.4%)	11 (39.3%)	3 (42.9%)	5 (21.7%)	6 (50.0%)
Leukocytosis at diagnosis	30 (21.6%)	16 (23.2%)	3 (10.7%)	3 (42.9%)	3 (13.0%)	5 (41.7%) ^g^

HIV: human immunodeficiency virus; ESRD: end-stage renal disease; CAPD: continuous ambulatory peritoneal dialysiData are presented as the median (interquartile range) for continuous data and percentages for categorical data

Defined as hemoglobin of 12 g/dl

Defined as a white blood cell count of 10,000/mm3

^a^: *P* < 0.05 Luminal TB vs. Peritoneal TB, ^b^: *P* < 0.05 Luminal TB vs. Nodal TB, ^c^: *P* < 0.05 Luminal TB vs. Visceral TB, ^d^: *P* < 0.05 Luminal TB vs. Mixed TB, ^e^: *P* < 0.05 Peritoneal TB vs. Nodal TB, ^f^: *P* < 0.05 Peritoneal TB vs. Visceral TB, ^g^: *P* < 0.05 Peritoneal TB vs. Mixed TB, ^h^: *P* < 0.05 Nodal TB vs. Visceral TB, ^i^: *P* < 0.05 Nodal TB vs. Mixed TB, ^j^: *P* < 0.05 Visceral TB vs. Mixed TB

Abdominal pain was the most common symptom (34.5%; Table 3). Incidence of abdominal distension was significantly higher in the peritoneal TB group than in the other 4 groups. However, there were no significant differences in the frequencies of other symptoms among the 5 study groups.

**Table 3:** Clinical features in 139 patients with abdominal tuberculosis

Characteristic	All patients (*n* = 139)	Luminal TB (*n* = 69)	Peritoneal TB (*n* = 28)	Nodal TB (*n* = 7)	Visceral TB (*n* = 23)	Mixed TB (*n* = 12)
Abdominal pain	48 (34.5%)	23 (33.3%)	7 (25.0%)	3 (42.9%)	9 (39.1%)	6 (50.0%)
Fever	16 (11.5%)	5 (7.2%)	6 (21.4%)	0 (0%)	3 (13.0%)	2 (16.7%)
Anorexia	9 (6.5%)	6 (8.7%)	1 (3.6%)	1 (14.3%)	0 (0%)	1 (8.3%)
Body weight loss	5 (3.6%)	4 (5.8%)	1 (3.6%)	0 (0%)	0 (0%)	0 (0%)
Abdominal distension	29 (21.0%)	2 (2.9%)	22 (78.6%) ^a, e, f, g^	0 (0%)	0 (0%)	5 (41.7%) ^d, j^
Bloody stool	11 (7.9%)	10 (14.5%)	0 (0%)	0 (0%)	1 (4.3%)	0 (0%)
Dyspnea	2 (1.4%)	0 (0%)	1 (3.6%)	0 (0%)	1 (4.3%)	0 (0%)
Diarrhea	6 (4.3%)	5 (7.2%)	0 (0%)	0 (0%)	0 (0%)	1 (8.3%)
Hematuria	1 (0.7%)	0 (0%)	0 (0%)	0 (0%)	1 (4.3%)	0 (0%)
Dysuria	2 (1.4%)	0 (0%)	0 (0%)	0 (0%)	2 (8.7%)	0 (0%)
Palpable mass	3 (2.2%)	1 (1.4%)	0 (0%)	1 (14.3%)	1 (4.3%)	0 (0%)
Scrotal swelling	1 (0.7%)	0 (0%)	0 (0%)	0 (0%)	1 (4.3%)	0 (0%)
Jaundice	1 (0.7%)	0 (0%)	0 (0%)	1 (14.3%)	0 (0%)	0 (0%)
No symptom	31 (22.3%)	23 (33.3%) ^a, d^	1 (3.6%)	2 (28.6%)	5 (21.7%)	0 (0%)

Data are presented as the median (interquartile range) for continuous data and percentages for categorical data

^a^: *P* < 0.05 Luminal TB vs. Peritoneal TB, ^b^: *P* < 0.05 Luminal TB vs. Nodal TB, ^c^: *P* < 0.05 Luminal TB vs. Visceral TB, ^d^: *P* < 0.05 Luminal TB vs. Mixed TB, ^e^: *P* < 0.05 Peritoneal TB vs. Nodal TB, ^f^: *P* < 0.05 Peritoneal TB vs. Visceral TB, ^g^: *P* < 0.05 Peritoneal TB vs. Mixed TB, ^h^: *P* < 0.05 Nodal TB vs. Visceral TB, ^i^: *P* < 0.05 Nodal TB vs. Mixed TB, ^j^: *P* < 0.05 Visceral TB vs. Mixed TB

### Diagnostic methods

In the 139 patients with abdominal TB, diagnosis was confirmed microbiologically in 51 patients (37.0%) and histologically in 59 patients (42.8%). Confirmed diagnosis was achieved in 76 patients (54.7%), while the remaining 63 patients (45.3%) were diagnosed clinically (Table 4). The frequency of microbiologic diagnosis (AFB smear or culture) was significantly higher in the visceral TB group (68.2%) than in the luminal (33.3%) and peritoneal (25.0%) TB groups. The frequency of histologic diagnosis (caseating granulomas on biopsy specimen) was significantly higher in the nodal TB group (85.7%) than in the luminal (39.1%) and peritoneal (28.6%) TB groups. The frequency of clinical diagnosis was significantly higher in the peritoneal TB group (64.3%) than in the nodal (14.3%) and visceral (13.6%) TB groups.

**Table 4:** Diagnostic method in 139 patients with abdominal tuberculosis

Characteristic	All patients (*n* = 139)	Luminal TB (*n* = 69)	Peritoneal TB (*n* = 28)	Nodal TB (*n* = 7)	Visceral TB (*n* = 23)	Mixed TB (*n* = 12)
Microbiologic diagnosis	51 (37.0%)	23 (33.3%)	7 (25.0%)	2 (28.6%)	15 (68.2%) ^c, f^	4 (33.3%)
Histological diagnosis	59 (42.8%)	27 (39.1%)	8 (28.6%)	6 (85.7%) ^b, e^	12 (54.5%)	6 (50.0%)
Clinical diagnosis	63 (45.3%)	35 (50.7%) ^c^	18 (64.3%) ^e, f^	1 (14.3%)	3 (13.6%)	6 (50.0%) ^j^
Operation	21 (15.1%)	2 (2.9%)	4 (14.3%)	3 (42.9%) ^b^	10 (43.5%) ^c, f^	2 (16.7%)
*Confirmed diagnosis by operation	19 (90.4%)	2 (100%)	4 (100%)	3 (100%)	9 (90%)	1 (50%)
Percutaneous biopsy group	16 (11.5%)	0 (0%) ^a, b, c, d^	6 (21.4%)	3 (42.9%)	3 (13.0%)	4 (33.3%)
*Confirmed diagnosis by percutaneous biopsy	13 (81.3%)	0/0	4 (66.7%)	2 (66.7%)	3 (100%)	4 (100%)
Endoscopy or colonoscopy	70 (50.4%)	65 (94.2%) ^a, b, c, d^	0 (0%)	0 (0%)	0 (0%)	4 (33.3%) ^g, j^
*Confirmed diagnosis by endoscopy or colonoscopy	34 (48.6%)	32 (49.2%)	0 (0%)	0 (0%)	0 (0%)	1 (25%)
Paracentesis	33	0	27 (96.4%) ^a, e, f, g^	0	0	6 (50.0%) ^g, j^
*Confirmed diagnosis by paracentesis	8 (24.2%)	0 (0%)	6 (21.4%) ^a,f^	0 (0%)	0 (0%)	2 (33.3%) ^d^

Data are presented as the median (interquartile range) for continuous data and percentages for categorical data

*A Confirmed diagnosis was defined as microbiologic or histological diagnosis

^a^: *P* < 0.05 Luminal TB vs. Peritoneal TB, ^b^: *P* < 0.05 Luminal TB vs. Nodal TB, ^c^: *P* < 0.05 Luminal TB vs. Visceral TB, ^d^: *P* < 0.05 Luminal TB vs. Mixed TB, ^e^: *P* < 0.05 Peritoneal TB vs. Nodal TB, ^f^: *P* < 0.05 Peritoneal TB vs. Visceral TB, ^g^: *P* < 0.05 Peritoneal TB vs. Mixed TB, ^h^: *P* < 0.05 Nodal TB vs. Visceral TB, ^i^: *P* < 0.05 Nodal TB vs. Mixed TB, ^j^: *P* < 0.05 Visceral TB vs. Mixed TB

Surgery was performed in 21 patients (15.1%) and provided a good diagnostic yield in 90.4% (19/21). Among them, 10 underwent surgery for diagnosis of suspected abdominal TB, and 11 for management of numerous complications, such as intussusception, abscess, perforation, obstruction, and hemorrhage. The frequency of surgery was higher in the nodal (42.9%) and visceral (43.5%) TB groups than in the luminal (2.9%) and peritoneal (14.3%) TB groups. Percutaneous biopsy was performed in 16 patients (11.5%); endoscopy or colonoscopy was performed in 70 patients (50.4%). Ascitic diagnosis through paracentesis was achieved in 33 patients (23.7%). Among them, a positive yield for AFB culture was observed in only 8 patients (24.2%). Diagnostic yield for confirmed diagnosis from surgery, percutaneous biopsy, endoscopy/colonoscopy, and paracentesis was 90.4, 81.3, 48.6, and 24.2%, respectively.

### Treatment outcomes

Of the 139 patients who underwent anti-TB treatment, 117 (84.2%) showed a complete response, 1 (0.7%) showed a partial response, 2 (1.4%) showed no response, 12 (8.6%) were lost to follow-up, 3 (2.2%) had recurrence, and 3 (2.2%) transferred out (Table 5). The frequency of complete response was significantly lower in the nodal TB group than in the other 4 study groups. Only 2 patients (1.4%) died during anti-TB therapy. One patient was positive for HIV and multidrug-resistant abdominal TB involving the liver, spleen, and genitourinary tract. The major cause of death in this patient was concomitant TB meningitis. Another non-HIV-positive patient died due to intestinal obstruction with sepsis.

**Table 5:** Treatment outcomes in 139 patients with abdominal tuberculosis

Characteristic	All patients (*n* = 139)	Luminal TB (*n* = 69)	Peritoneal TB (*n* = 28)	Nodal TB (*n* = 7)	Visceral TB (*n* = 23)	Mixed TB (*n* = 12)
Duration of treatment, days	194.0 (176.0–258.0)	189.0 (175.0–219.0)	200.0 (165.0–258.0)	258.0 (16.0–285.0)	284.0 (178.0–284.0)	245.0 (177.5–349.0)
Side effect	21 (15.2%)	11 (15.9%)	3 (11.1%)	0 (0%)	4 (17.4%)	3 (25.0%)
Drug-induced liver injury	9 (6.5%)	5 (8.7%)	2 (7.1%)	0 (0%)	0 (0%)	1 (8.3%)
Nephrectomy	4 (2.9%)	0 (0%)	0 (0%)	0 (0%)	4 (17.4%) ^c, f^	0 (0%)
First-line anti-TB drug	131 (94.2%)	66 (95.7%)	27 (96.4%)	7 (100%)	20 (87.0%)	11 (91.7%)
Second-line anti-TB drug	8 (5.8%)	3 (4.3%)	1 (3.6%)	0 (0%)	3 (13.0%)	1 (8.3%)
Complete response	117 (84.2%)	62 (89.9%)	22 (78.6%)	3 (42.9%) ^b, e, h, i^	20 (87.0%)	10 (83.3%)
Partial response	1 (0.7%)	0 (0%)	0 (0%)	1 (14.3%)	0 (0%)	0 (0%)
No response	2 (1.4%)	1 (1.4%)	0 (0%)	0 (0%)	1 (4.3%)	0 (0%)
Lost to follow up	12 (8.6%)	4 (5.8%)	5 (17.9%)	0 (0%)	1 (4.3%)	2 (16.7%)
Recurrence	3 (2.2%)	1 (1.4%)	1 (3.6%)	1 (14.3%)	0 (0%)	0 (0%)
Transfer out	3 (2.2%)	1 (1.4%)	0 (0%)	2 (28.6%)	0 (0%)	0 (0%)
Death	2 (1.4%)	1 (1.4%)	0 (0%)	0 (0%)	1 (4.3%)	0 (0%)

Data are presented as the median (interquartile range) for continuous data and percentages for categorical data

^a^: *P* < 0.05 Luminal TB vs. Peritoneal TB, ^b^: *P* < 0.05 Luminal TB vs. Nodal TB, ^c^: *P* < 0.05 Luminal TB vs. Visceral TB, ^d^: *P* < 0.05 Luminal TB vs. Mixed TB, ^e^: *P* < 0.05 Peritoneal TB vs. Nodal TB, ^f^: *P* < 0.05 Peritoneal TB vs. Visceral TB, ^g^: *P* < 0.05 Peritoneal TB vs. Mixed TB, ^h^: *P* < 0.05 Nodal TB vs. Visceral TB, ^i^: *P* < 0.05 Nodal TB vs. Mixed TB, ^j^: *P* < 0.05 Visceral TB vs. Mixed TB

**Fig. 2:**
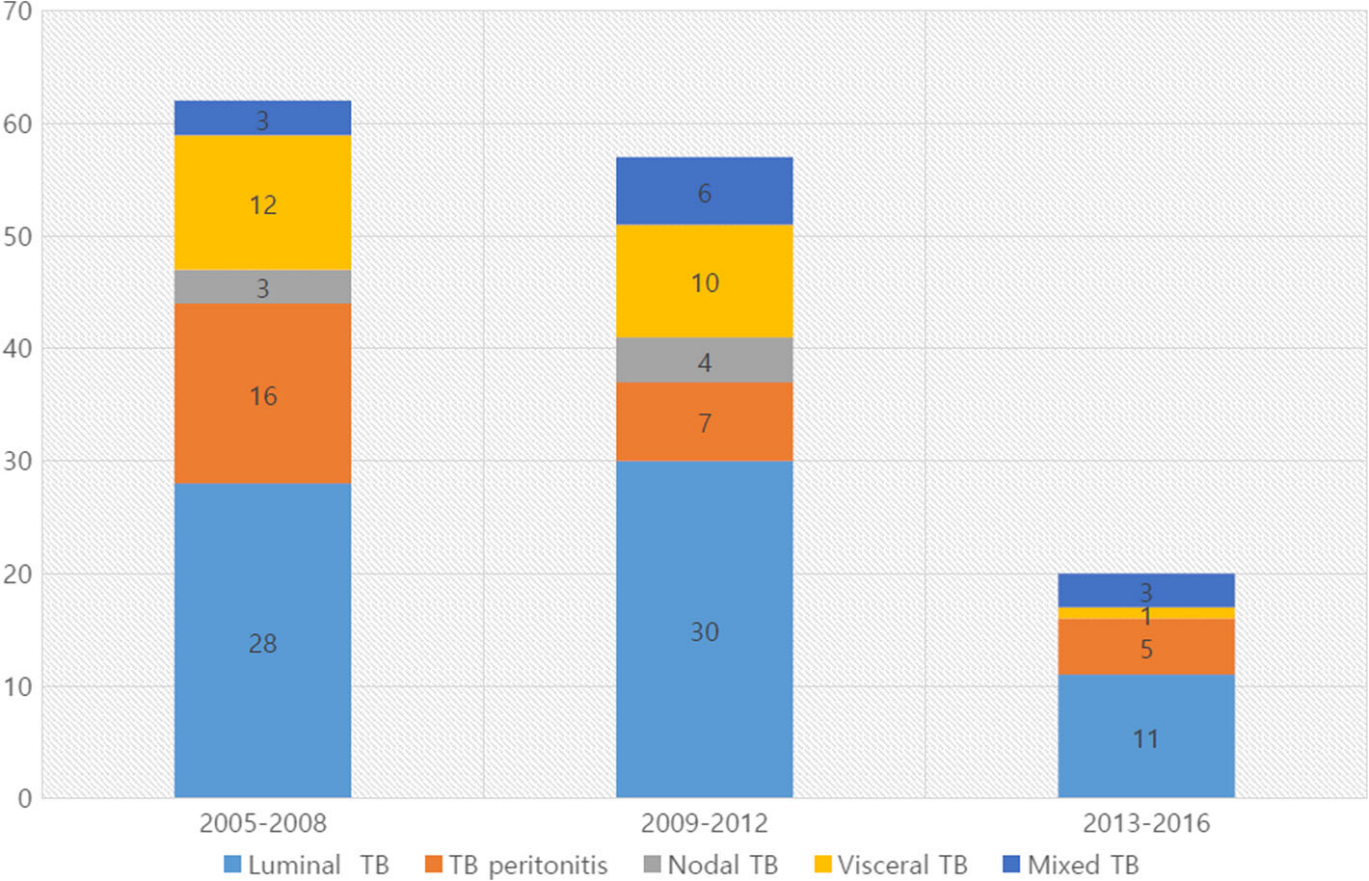
Cases of abdominal tuberculosis (TB), 2005–2008, 2009–2012, and 2013–2016, respectively. In 2010, incidence of abdominal TB tended to decrease

Twenty-one patients (15.2%) had adverse effects due to anti-TB agents, and 9 (6.5%) developed drug-induced liver injury. The median duration of anti-TB treatment was 194 days; 131 patients were treated with first-line anti-TB agents, while 8 received second-line anti-TB agents. Figure 2 shows an overall decrease of approximately 60% in number of TB cases in southeastern Korea in the period 2005–2008 to 2013–2016.

## Discussion

In the current study, we described the distribution of abdominal TB at a tertiary hospital in southeastern Korea. The most frequent site of abdominal TB was the luminal tract (49.6%) followed by the peritoneum (20.1%), solid viscera (16.5%), mixed organs (8.6%), and lymph nodes (5.0%). The most common presentation was abdominal pain (34.5%), whereas abdominal distension was a unique presentation in patients with peritoneal TB. According to diagnostic method, the frequency of clinical diagnosis was highest in the luminal and peritoneal TB groups, while the frequency of microscopic diagnosis was highest in the visceral TB group, and the frequency of histologic diagnosis was highest in the nodal TB group. Interestingly, 117 patients (84.2%) showed a complete response and the mortality rate was only 1.4%.

Abdominal TB poses a considerable diagnostic challenge due to the lack of specific symptoms and pathognomonic findings. Moreover, no single diagnostic method is sufficient for diagnosis. Based on our findings, colonoscopy and paracentesis may be useful in cases of luminal and peritoneal TB, where mucosal or peritoneal lesions are accessible. However, colonoscopy and peritoneal fluid analysis have a low diagnostic yield for confirmed diagnosis of abdominal TB (49.2 and 22.2%, respectively). Therefore, the majority of luminal and peritoneal TB cases were diagnosed based on clinical response to anti-TB agents and radiologic findings. Among these, laparotomy was performed in only 2 and 4 patients in the luminal and peritoneal TB groups, respectively. Peritoneal fluid analysis is the most useful nonoperative diagnostic method for peritoneal TB. High ascitic adenosine deaminase activity level (> 33 IU/L) and low serum ascitic albumin gradient (< 1.1) have a sensitivity of 97% and specificity of 100% [17]. Therefore, laparoscopy could be used to rule out other intra-abdominal malignancies and to minimize any possible diagnostic delay in these groups. On the other hand, diagnosis of nodal TB was confirmed from surgical procedures or percutaneous biopsy in 6 patients (confirmed diagnosis), while 1 patient was diagnosed based on clinical response to anti-TB agents and computed tomography imaging (clinical diagnosis). Diagnosis of visceral TB was confirmed from surgical procedures or percutaneous biopsy in 13 patients, and from urine AFB in 7 patients (confirmed diagnosis), while 3 patients were diagnosed based on clinical response to anti-TB agents and computed tomography imaging (clinical diagnosis). Although nodal and visceral TB are mainly treatable medically, surgery is still often required for suspected abdominal TB and management of complications, such as infection, perforation, and hemorrhage.

A 6-month course of anti-TB therapy for luminal TB is recommended in treatment guidelines [18, 19]. Two previous prospective, randomized studies confirmed a high cure rate of > 90% after both 6 and 9 months of standard anti-TB therapy [6, 16]. In addition, some retrospective studies have shown that anti-TB agents are usually highly effective and associated with low mortality (0–6%) in abdominal TB [7, 8, 13, 20, 21]. However, abdominal TB has a high mortality rate (6–20%), and the majority of patients had acute complications and required emergency exploratory laparotomy in several other studies [22–25]. In our study population, most patients with abdominal TB showed a good response to anti-TB agents and had a good prognosis. Moreover, only 3 cases relapsed and required additional treatment, and the mortality rate was only 1.4%, suggesting that if diagnosed early, abdominal TB can be treated successfully with anti-TB agents, unlike high-mortality study groups. However, nodal TB is difficult to treat based on the low rate of complete response (42.9%), and surgery is often still required for suspected diagnosis.

Drug-induced liver injury during anti-TB treatment is the most common reason leading to discontinuation of therapy [26]. In addition, hepatitis B and C virus are major risk factors for liver injury [27, 28]. We noted drug-induced liver injury in 9 patients (6.5%), including 2 with hepatitis B and 1 with hepatitis C. In 8 patients, all drugs were stopped and a step-wise rechallenge was initiated after complete resolution of hepatotoxicity; 1 patient died due to intestinal obstruction.

The limitations of this study include its single-center, retrospective nature, relatively small sample size, especially in the nodal TB group, and incomplete diagnostic confirmation in all patients. In addition, precise information on alcohol consumption, underlying disease, and drug compliance were unavailable from medical record review. Our study has a low frequency of mixed TB compared to previous studies [14]. Because of the retrospective nature of this study, computed tomography was not performed in all patients with ascites. This is the major limitation of this study. Thus, further prospective studies on abdominal TB including more patients are required.

## Conclusion

Abdominal TB can be of various forms, including luminal, peritoneal, nodal, and visceral. A high index of clinical suspicion is required to make a diagnosis of abdominal TB due to the nonspecific clinical symptoms and radiologic features. Early diagnosis with prompt treatment is essential for a promising prognosis. Most cases respond well to medical therapy, and surgery is required in only a minority of cases.

## Abbreviations

AFB: acid-fast bacilli
TB: tuberculosis
